# Physician perceptions of drug utilization management: Results of a national survey

**DOI:** 10.1371/journal.pone.0274772

**Published:** 2022-09-20

**Authors:** Marie Louise Edwards, Perry T. Yin, Michael Kuehn, Keith Bratti, Noam Kirson, Anupam B. Jena, Scott Howell

**Affiliations:** 1 Analysis Group, Inc, New York, New York, United States of America; 2 US Pharmaceuticals, Novartis Pharmaceuticals Corporation, East Hanover, New Jersey, United States of America; 3 Analysis Group, Inc, Boston, Massachusetts, United States of America; 4 Department of Health Care Policy, Harvard Medical School, Boston, Massachusetts, United States of America; Khon Kaen University Faculty of Pharmaceutical Sciences, THAILAND

## Abstract

The use of drug utilization management techniques such as formulary exclusions, prior authorizations, and step edits has risen sharply during the last decade, contributing to the growing burden on physicians and patients. Limited quantitative data exist, however, on physician perceptions of drug utilization management. A national survey was conducted between February 9 and March 30, 2021, targeting office-based physicians working in the United States to assess their perceptions on drug utilization management in their practice. Of the 742 physicians that participated in the study, over 80% reported deciding against prescribing certain treatments in anticipation of drug utilization management at least sometimes (>50% of the time). Despite utilization management having an impact on prescribing decisions, about half of physicians said that the utilization management policies they encounter rarely or never (0–25% of the time) align with clinical evidence.

## Introduction

Drug utilization management is designed to ensure patient safety and the use of cost-effective medicines. However, its use has risen sharply during the last decade, placing increasing burden on physicians and patients [1–3]. For instance, the largest three pharmacy benefit managers expanded formulary exclusions from 109 drugs in 2014 to 846 in 2020 [4]. Similarly, one-third of large commercial payers now impose utilization management on specialty drugs that are more stringent than those on the Food and Drug Administration’s label [5].

While previous studies have sought to quantify costs incurred by physician practices due to payer interactions as well as time spent on administration, there has been less of a focus on physician perceptions of drug utilization management [6–17]. As a first step towards filling this gap, we conducted an online survey of US-based physicians, focusing on the impact of drug utilization management policies on physician practices, perceived alignment of such policies with clinical guidelines, and preferred evidence for informing such policies.

## Methods

A survey targeting US physicians was developed to assess the impact of drug utilization management on physician practices. This instrument was designed to capture information on drug utilization management related to retail prescriptions and physician-administered medications, including prior authorizations, formulary restrictions, step edits, and other or unknown payer policies. The survey instrument development was informed by a targeted literature search. Following initial survey development, the survey instrument was further refined based on three pre-test interviews with an office administrator, a primary care physician and a specialist physician. The survey included questions about respondent and practice characteristics such as years of experience, practice specialty, and prescription volume, impact of drug utilization management policies on physician practices, perceived alignment of such policies with clinical guidelines, and preferred evidence for informing such policies.

The survey was fielded online to a large US-based panel maintained by M3 Global Research between February 9 and March 30, 2021. M3’s panel includes physicians from all US states, with a distribution of age, gender, and practice type that is similar to the physicians registered with the American Medical Association (AMA). Physicians were required to be licensed to practice medicine in the US and to be working at primary care or select specialist outpatient practices. Specialist practices included those the authors considered likely to face drug utilization management: allergy & immunology, cardiovascular disease, dermatology, endocrinology, family medicine/general practice, gastroenterology, geriatric medicine, oncology & hematology, internal medicine, nephrology, neurology, ophthalmology, pain medicine & pain management, psychiatry, pulmonary disease, rheumatology, and urology. Physicians working in specialties unlikely to encounter frequent drug utilization management and those working in the emergency room or inpatient settings were excluded. Further, those working in outpatient military clinics, Veteran Affairs centers, or other government hospitals were removed due to their unique payer coverage policies. Recruitment quotas were established based on the practice size and type (i.e., PCP vs. specialist) to obtain a balanced distribution. Practice size was defined by the number of physicians working at the practice (small [1−5 physicians], medium [6−19 physicians], large [20+]) and these three groups were targeted to ensure that each would make up roughly a third of the sample. The recruitment quota for practice type was evenly divided by primary care and specialist, to ensure that a variety of perspectives and experiences related to UM were included in the sample, as UM may impact these types differently.

Physicians were compensated at fair market value rates for completing the survey. Participants were informed about the aim of the study and written consent from the participants was required to start the survey. Study participant consent covered compliance with adverse event and product complaint reporting if applicable, processing of personal data for the purposes of the study, and obtaining applicable permissions to take part in the study e.g., if required by an employer. It should be noted that the study did not collect personal information and respondent information was anonymized at data collection.

This study was entirely descriptive; no hypothesis testing was conducted. We did not seek the approval of an institutional review board as the study was based on primary market research of office-based physicians and did not involve any patient-level data. Outliers for key outcome variables were removed from the data based on a threshold of three times the standard deviation above the mean. Mean, median, and standard deviation (SD) were used to summarize continuous variables; counts, frequencies, and percentages were used to summarize categorical variables. It was not possible to estimate the margin of error as our sample was non-random. All analyses were performed in R, version 3.6.3, and SAS, version 9.4 (with more detail on the analyses provided in S1 Appendix).

## Results

A total of 22,648 physicians were invited to complete the survey. Of those invited, 1,887 (8.3%) answered the survey eligibility questions. Among those who answered the eligibility questions, 881 (46.7%) met the criteria to be included in the study. Of the eligible respondents, 742 (84.2%) completed the survey and were included in the final sample.

Per the study design, the sample was evenly divided by practice size, and between respondents working in primary care and one of 15 other specialties. Primary care physicians (N = 369) worked in family medicine/general practice (75.9%) and internal medicine (24.1%) offices. Among specialist physicians (N = 373), the most common specialist types were ophthalmology (19.3%), dermatology (13.9%), and endocrinology (9.1%) **(**Table 1**)**. A median of nine physicians were employed at the surveyed practices. The majority of physicians (69.4%) worked in private practice and treated privately insured (47.9%) or Medicare (including Medicare Advantage) (31.2%) patients. Lastly, physicians reported seeing a median of 100 patients in a typical week.

**Table 1:** Respondent, practice, and prescription characteristics.

	Physicians N = 742
**Respondent characteristics**	
** Number of years in practice**	
** **Mean (SD)	17.5 (9.7)
** **Median	18.0
** Specialty, N (%)**	
** **Primary care	369 (49.7%)
** **Specialty medicine	373 (50.3%)
**Practice characteristics**	
** Practice size, N (%)**	
** **Small: 1–5 physicians	256 (34.5%)
** **Medium: 6–19 physicians	259 (34.9%)
** **Large: ≥20 physicians	227 (30.6%)
** Primary practice type, N (%)**	
** **Private practice	515 (69.4%)
** **Community-based clinic	119 (16.0%)
** **Academic institution	98 (13.2%)
** **Hospital or hospital-owned (not VA or government)	92 (12.4%)
** Geographic region, N (%)**	
** **Midwest	172 (23.2%)
** **Northeast	166 (22.4%)
** **South	245 (33.0%)
** **West	159 (21.4%)
** Percentage breakdown of patient insurance types, mean (SD)**	
** **Private, commercial	47.9 (20.5)
** **Medicare (including Medicare Advantage, Part B, Part D, etc.)	31.2 (15.4)
** **Medicaid	13.5 (14.7)
** **Uninsured	4.5 (6.9)
** **Other (e.g., VA, military)	2.9 (6.9)
**Prescription characteristics**	
** Number of drug prescriptions in a typical week**	
** **Mean (SD)	155.1 (159.4)
** **Median	100.0
** Percentage breakdown of drug prescription by type, mean (SD)**	
** **Generic drugs	59.3 (21.4)
** **Branded drugs	25.4 (15.0)
** **Specialty drugs	10.1 (11.2)
** **Physician-administered drugs	5.2 (9.6)
** Percentage of prescriptions subject to each type of drug utilization management, mean (SD)** ^**1**^	
** **Formulary restrictions	19.4 (19.9)
** **Prior authorizations	18.7 (20.7)
** **Step edits	9.6 (13.3)
Other or unknown types of drug utilization management	6.2 (11.2)

Abbreviations: SD: Standard deviation; VA: Veterans Affairs.

^1^Of the 742 surveyed physician respondents, N = 722 (97%) provided answers to the questions about the frequency of four types of drug utilization management; N = 20 (3%) physician respondents responded that they did not know their volumes of drug utilization management, so they were excluded from these summary statistics.

On average, physicians reported handling 155 drug prescriptions for their patients per week. Prior authorizations and formulary restrictions, reported to impact roughly one in five prescriptions on average, were more common than step edits and other or unknown types of drug utilization management (impacting ≤10% of prescriptions). Overall, physicians reported weekly average volumes of 23.5 formulary restrictions, 19.7 prior authorizations, 12.6 step edits, and 7.9 other types of drug utilization management.

Among surveyed physicians, 82.3% reported deciding against prescribing certain treatments in anticipation of drug utilization management at least sometimes (>50% of the time) (Table 2). Moreover, drug utilization management at least sometimes resulted in longer patient visits (67.1% of respondents), additional patient visits (51.9%), and additional lab tests or imaging (55.0%). As a result, physicians reported spending extra time working (67.7%), spending less time on other administrative tasks (30.1%), scheduling fewer patient visits (16.2%), and rescheduling patient visits (12.1%).

**Table 2:** Physician perceptions of drug utilization management.

	Physicians N = 742
**Perceived impact of drug utilization management**	
** Frequency of deciding against prescribing a treatment due to drug utilization management, N (%)**	
** **Always (100% of the time)	49 (6.6%)
** **Usually (~75% of the time)	254 (34.2%)
** **Sometimes (~50% of the time)	308 (41.5%)
** **Rarely (~25% of the time)	127 (17.1%)
** **Never (0% of the time)	4 (0.5%)
** Frequency of patient visits lasting longer due to drug utilization management discussions**	
** **Always (100% of the time)	27 (3.6%)
** **Usually (~75% of the time)	164 (22.1%)
** **Sometimes (~50% of the time)	307 (41.4%)
** **Rarely (~25% of the time)	231 (31.1%)
** **Never (0% of the time)	13 (1.8%)
** Frequency of patients coming in for additional visits due to drug utilization management**	
** **Always (100% of the time)	13 (1.8%)
** **Usually (~75% of the time)	88 (11.9%)
** **Sometimes (~50% of the time)	284 (38.3%)
** **Rarely (~25% of the time)	306 (41.2%)
** **Never (0% of the time)	51 (6.9%)
** Frequency of drug utilization management causing patients to have additional lab tests or imaging**	
** **Always (100% of the time)	24 (3.2%)
** **Usually (~75% of the time)	94 (12.7%)
** **Sometimes (~50% of the time)	290 (39.1%)
** **Rarely (~25% of the time)	286 (38.5%)
** **Never (0% of the time)	48 (6.5%)
** Additional consequences of drug utilization management in a typical week, N (%)** ^**1**^	
** **Had to spend extra time working	502 (67.7%)
** **Had to cut down the amount of time spent on other administrative tasks	223 (30.1%)
** **Had to schedule fewer patient visits	120 (16.2%)
** **Had to reschedule patient visits	90 (12.1%)
** **None of the above	136 (18.3%)
**Frequency of drug utilization management aligning with clinical guidelines, N (%)**	
** Formulary restrictions**	
** **Always (100% of the time)	22 (3.1%)
** **Usually (~75% of the time)	105 (14.9%)
** **Sometimes (~50% of the time)	228 (32.4%)
** **Rarely (~25% of the time)	265 (37.7%)
** **Never (0% of the time)	83 (11.8%)
** Prior authorizations**	
** **Always (100% of the time)	28 (3.9%)
** **Usually (~75% of the time)	92 (13.0%)
** **Sometimes (~50% of the time)	214 (30.2%)
** **Rarely (~25% of the time)	291 (41.0%)
** **Never (0% of the time)	84 (11.8%)
** Step edits**	
** **Always (100% of the time)	17 (2.8%)
** **Usually (~75% of the time)	91 (15.1%)
** **Sometimes (~50% of the time)	189 (31.4%)
** **Rarely (~25% of the time)	228 (37.9%)
** **Never (0% of the time)	77 (12.8%)
**Preferred evidence to inform medically justified drug utilization management, N (%)** ^**1**^	
** **Medical society guidelines (e.g., NCCN guidelines, compendia listings)	494 (66.6%)
** **Clinical trial data (e.g., trial results, meta-analyses, or systematic reviews)	466 (62.8%)
** **Real world outcomes evidence	408 (55.0%)
** **FDA labels	231 (31.1%)
** **Payer’s discretion	88 (11.9%)
** **None of the above	58 (7.8%)

Abbreviations: FDA: Food and Drug Administration; NCCN: National Comprehensive Cancer Network.

^1^Respondents could select more than one option; results are not mutually exclusive.

When asked how often drug utilization management aligns with clinical guidelines, about half of physicians said that formulary restrictions, prior authorizations, and step edits rarely or never (0–25% of the time) align with clinical evidence (Table 2). Among the types of evidence that physicians said ought to be used to inform drug utilization management, 66.6% prioritized medical society guidelines, 62.8% said clinical trial data, 55.0% said real-world outcomes evidence, 31.1% referenced the FDA label, and 11.9% preferred payer’s discretion.

## Discussion

Drug utilization management plays an important role in insurance benefit design. By encouraging generic and therapeutic substitution, these techniques can save patients hundreds of dollars each year [18]. Studies have also shown that it can reduce the number of adverse drug events and improve safety and outcomes for patients [19]. However, although drug utilization management is designed to ensure patient safety and the use of cost-effective medicines, it does impact physician practices. Physicians and their staff are spending increasing time on tasks related to drug utilization management, including interacting with payers [11,17]. Beyond time spent directly on these tasks, our study indicates that drug utilization management not only impacts treatment decisions, it also leads to longer and additional patient visits as well as more testing and imaging.

Critically, most physicians reported that the current implementation of drug utilization management is frequently misaligned with clinical evidence. This is consistent with a 2020 American Medical Association study in which 32% of surveyed physicians reported that prior authorization criteria are rarely or never evidence-based [13]. Instead, physicians in our study indicated that drug utilization management policies should draw more from medical society guidelines, clinical trial data, and real-world outcomes.

This suggests that there is a need for the industry to define and implement principles for drug utilization management policies with the aim to ensure that these policies align more closely with clinical evidence. To this end, several stakeholder-driven groups–the Institute for Clinical and Economic Review (ICER), American Medical Association (AMA), Academy of Managed Care Pharmacy (AMCP) and National Pharmaceutical Council (NPC)–have recently proposed criteria with which to define value-based patient access [20–23]. While this is a good first step in moving the conversation in the right direction, these efforts and the practical implementation of such criteria remain in its infancy.

This study should be considered in the context of its limitations. First, it focused on a subset of physician specialties. Moreover, the distribution of specialists was dependent on M3’s panel and those who met our inclusion criteria, as no sub-quotas were set for the specialist distribution. As a result, our findings may not be generalizable to all specialties. Second, it should be noted that several of the variables had high standard deviations. This suggests that a small number of respondents entered high values, which is tied to both recall bias and the variation in respondent and practice characteristics included in the sample (i.e., physician from very large practices reported the maximum number of prescriptions per week). As such, our results should be considered in the context of the variation of practice types included in the sample. Third, given the fact that the physicians invited to the survey could choose whether or not to participate in the study, there is the risk of self-selection bias. Lastly, the study was conducted during the COVID-19 pandemic. Additionally, as with all analyses of survey data, this study may be affected by other potential biases, including recall bias (e.g., unknown, not sure response options) and non-random missing data (e.g., specifically omitting a particular answer option across questions).

Given the wide range of types of practices and the corresponding physician experiences with utilization management, future research should further evaluate whether differences in practice types (i.e., physician group size or integration with a health system) and physician characteristics (i.e., physician age and length of experience) impacts physicians’ perceptions of drug utilization management.

## Conclusion

Drug utilization management impacts physician treatment decisions and leads to additional resource use by patients. Critically, a majority of physicians report that these policies rarely or never align with clinical guidelines. As the prevalence of drug utilization management continues to grow, the impact on physician treatment decisions and subsequently patients will continue to be an important topic.

## Supporting information

S1 Appendix (DOCX)
