# Supplementary material for: Physician perceptions of drug utilization management: Results of a national survey
Source: PLoS One. 2022 Sep 20;17(9):e0274772. doi: 10.1371/journal.pone.0274772 (PMC9488785; doi:10.1371/journal.pone.0274772)
Supplement: S1 Appendix — (DOCX) [file pone.0274772.s001.docx]

**Physician perceptions of drug utilization management: results of a national survey**

**APPENDIX**

## 1. Sample detail

*Appendix Exhibit 1. Inclusion flowchart*


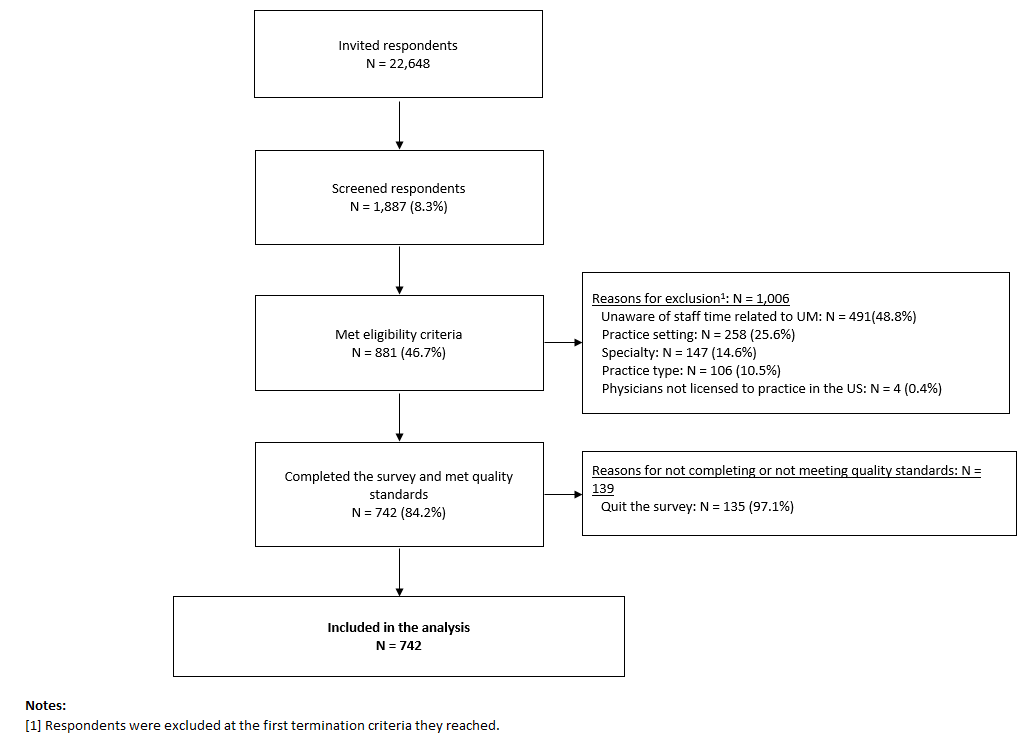


### 1.1. Practice distribution

Appendix Exhibit 1 summarizes the practice specialties of the surveyed respondents.

**Appendix Exhibit 1. Distribution of respondents’ practice specialties**

|  | **Physician** | |
| --- | --- | --- |
|  | **N = 742** | |
| **Practice specialty, N (%)** |  |  |
| **Primary care/general medicine** | **369** | **(49.7%)** |
| Family Medicine/General Practice | 280 | (75.9%) |
| Internal Medicine | 89 | (24.1%) |
| **Specialty medicine** | **373** | **(50.3%)** |
| Allergy & Immunology | 22 | (5.9%) |
| Cardiovascular Disease | 27 | (7.2%) |
| Dermatology | 52 | (13.9%) |
| Endocrinology, Diabetes & Metabolism | 34 | (9.1%) |
| Gastroenterology | 33 | (8.8%) |
| Geriatric Medicine | 1 | (0.3%) |
| Nephrology | 17 | (4.6%) |
| Neurology | 17 | (4.6%) |
| Oncology | 20 | (5.4%) |
| Ophthalmology | 72 | (19.3%) |
| Pain Medicine & Pain Management | 14 | (3.8%) |
| Psychiatry | 24 | (6.4%) |
| Pulmonary Disease | 11 | (2.9%) |
| Rheumatology | 16 | (4.3%) |
| Urology | 13 | (3.5%) |

## 2. Physician panel characteristics

The study respondents were recruited from a national physician panel covering all states of the US and with a similar distribution of age, gender, and practice type as the physicians registered with the American Medical Association (AMA): 30% females in the panel versus 32% in AMA, 70% age 35-64 versus 64% in AMA; 11% hospital-based versus 11% in AMA; and 63% office-based versus 58% in AMA.

The sample was comparable to the US nationwide in terms of weekly patients seen by physicians (100 in our study vs. 101 in the US^1^); region (22.8% Midwest, 23.0% Northeast, 33.9% South, 20.2% West in our study vs. 20.7% Midwest, 17.2% Northeast, 38.3% South, 23.7% West^2^); and location (54.8% suburban in our study vs. 52.0% suburban in the US^3^). In terms of patient insurance breakdown, our sample reported 46.7% privately insured patients vs. 66.5% private in the US^4^.

## 3. Respondent compensation

Study participants received the following compensation: specialist physicians $51 and primary care physicians $40.

## 4. Data

The data underlying the results presented in the study are available in the following file:

## 5. References

1. 2018 Survey of America's Physicians: Practice Patterns & Perspectives. Internet. The Physicians Foundation. Jan 13, 2022. <https://physiciansfoundation.org/wp-content/uploads/2018/09/physicians-survey-results-final-2018.pdf>

2. US Census Bureau United States Population Growth by Region. Internet. US Census Bureau. Jan 13, 2022. <https://www.census.gov/popclock/print.php?component=growth&image=//www.census.gov/popclock/share/images/growth_1561939200.png>

3. The 2017 AHS Neighborhood Description Survey. Internet. US Department of Housing and Urban Development. Jan 13, 2022. <https://www.huduser.gov/portal/AHS-neighborhood-description-study-2017.html>

4. Keisler-Starkey K, Bunch LN. Health Insurance Coverage in the United States: 2020. Internet. US Census Bureau. Jan 13, 2022. <https://www.census.gov/library/publications/2021/demo/p60-274.html>

## 6. Survey instrument

|  | **US physicians**  (Q1 = 1) | | | |
| --- | --- | --- | --- | --- |
|  | **Practice size** | | |  |
|  | *Small* | *Medium* | *Large* | **Total** |
| # of physicians | 1- 5  (Q3 ≤ 5) | 6-19  6 ≤ Q3 ≤19) | 20+  (Q3≥ 20+) |  |
| **Sample size** |  |  |  |  |
| **Primary care**  (Q4 = 10 or 16) | 125 | 125 | 100 | 350 |
| **Specialists**  (Q4 ≠ 10, 14 or 16) | 115 | 115 | 100 | 330 |
| **Oncologists***  (Q4 = 14) | (no hard quota for practice size) | | | 20 |
| **Total** |  |  |  | **700** |

* 20 oncologists from any practice size.

**Introduction**

Welcome and thank you for participating in this study. This study aims to assess the impact on

your practice caused by payer drug coverage policies related to prescription and physician-administered medications. Payer drug coverage policies include formulary restrictions, prior authorizations and step edits.

Please be assured that the personal information you provide will be kept confidential. This is a double-blind study, and no selling or solicitation will result from your participation in this study.

If you get disconnected, please use the original link sent in the email invitation/reminder and you will be reconnected to the survey. If you have any other questions or suggestions, please contact us by replying to the email sent to you, and include the original text sent.

We will proceed with the first section, Screener.

Section A: Screener [SHOW TO RESPONDENTS AT THE TOP OF Q1]

Q1. What is your current position? [SINGLE-SELECT]

| 🔿 | 1. Physician [If participant selects Q1 = 1 and 700 completed participants already selected Q1 = 1, then the survey will be terminated immediately] |
| --- | --- |
| 🔿 | 4. Nurse practitioner, physician assistant, or other clinical staff [If participants selects Q1 = 4 and 200 completed participants already selected Q1 = 2 or Q1 = 4, then the survey will be terminated immediately] |
|  | 1a. [If participants selects Q1 = 4 then prompt Q1a]  Do you have administrative responsibilities at your primary practice?  1. Yes  2. No [If participant selects Q1a = 2, then the survey will be terminated immediately] |
|  | 1ai. [If participants selects Q1a = 1 then prompt Q1ai]  What proportion of your time is spent on administrative responsibilities?  _______% [0 - 100] [If Q1ai < 50, then the survey will be terminated immediately] |
| 🔿 | 2. Practice administrator, office manager, ~~or~~ practice manager, or medical manager [If participants selects Q1 = 2 and 200 completed participants already selected Q1 = 2 or Q1 = 4, then the survey will be terminated immediately] |
| 🔿 | 3. Other, please specify ________[OE] [If participant selects Q1 = 3, then the survey will be terminated immediately] |

[If the participant is a physician (Q1 = 1), then question Q2 should be prompted]

Q2. Are you licensed to practice medicine in the US? [SINGLE-SELECT]

| 🔿 | 1. Yes |
| --- | --- |
| 🔿 | 2. No [If participant selects Q2 = 2, then the survey will be terminated immediately] |

Q3. Approximately how many physicians are employed at your primary practice? [NUMERIC]

*Please enter a whole number*

__________ [1- 500]

[If the participant is a physician (Q1 = 1), then question ⁠Q4a should be prompted]

[If the participant is an administrator (Q1 = 2 or Q1 = 4), then question Q4b should be prompted]

Q4. a. Please indicate your primary specialty. [SINGLE-SELECT]

b. Please indicate the primary practice area of your office. [SINGLE-SELECT]

| 🔿 | 1. Allergy & Immunology |
| --- | --- |
| 🔿 | 2. Anatomic/Clinical Pathology [If participant selects Q4 = 2, then the survey will be terminated immediately] |
| 🔿 | 3. Anesthesiology  [If participant selects Q4 = 3, then the survey will be terminated immediately] |
| 🔿 | 4. Cardiovascular Disease |
| 🔿 | 5. Child & Adolescent Psychiatry [If participant selects Q4 = 5, then the survey will be terminated immediately] |
| 🔿 | 6. Critical Care Medicine [If participant selects Q4 = 6, then the survey will be terminated immediately] |
| 🔿 | 7. Dermatology |
| 🔿 | 8. Emergency Medicine [If participant selects Q4 = 8, then the survey will be terminated immediately] |
| 🔿 | 9. Endocrinology, Diabetes & Metabolism |
| 🔿 | 10. Family Medicine/General Practice |
| 🔿 | 11. Gastroenterology |
| 🔿 | 12. General Surgery  [If participant selects Q4 = 12, then the survey will be terminated immediately] |
| 🔿 | 13. Geriatric Medicine |
| 🔿 | 14. Hematology & Oncology  [If participant is an administrator (Q1 = 2 or Q1 = 4) and selects Q4 = 14, then the survey will be terminated immediately;  If participant is a physician (Q1 = 1) and selects Q4 = 14 and ≥ 20 completed surveys for physicians with Q4 = 14, then the survey will be terminated immediately] |
| 🔿 | 15. Infectious Disease [If participant selects Q4 = 15, then the survey will be terminated] |
| 🔿 | 16. Internal Medicine |
| 🔿 | 17. Internal Medicine/Pediatrics [If participant selects Q4 = 17, then the survey will be terminated immediately] |
| 🔿 | 18. Interventional Cardiology [If participant selects Q4 = 18, then the survey will be terminated immediately] |
| 🔿 | 19. Neonatal-Perinatal Medicine [If participant selects Q4 = 19, then the survey will be terminated immediately] |
| 🔿 | 20. Nephrology |
| 🔿 | 21. Neurological Surgery  [If participant selects Q4 = 21, then the survey will be terminated immediately] |
| 🔿 | 22. Neurology |
| 🔿 | 23. Neuroradiology  [If participant selects Q4 = 23, then the survey will be terminated immediately] |
| 🔿 | 24. Obstetrics & Gynecology [If participant selects Q4 = 24, then the survey will be terminated immediately] |
| 🔿 | 25. Ophthalmology |
| 🔿 | 26. Orthopedic Surgery  [If participant selects Q4 = 26, then the survey will be terminated immediately] |
| 🔿 | 27. Otolaryngology [If participant selects Q4 = 27, then the survey will be terminated immediately] |
| 🔿 | 28. Pain Medicine & Pain Management |
| 🔿 | 29. Pediatric Cardiology [If participant selects Q4 = 29, then the survey will be terminated immediately] |
| 🔿 | 30. Pediatric Hematology/Oncology  [If participant selects Q4 = 30, then the survey will be terminated immediately] |
| 🔿 | 31. Pediatrics [If participant selects Q4 = 31, then the survey will be terminated immediately] |
| 🔿 | 32. Physical Medicine & Rehabilitation [If participant selects Q4 = 32, then the survey will be terminated immediately] |
| 🔿 | 33. Plastic Surgery  [If participant selects Q4 = 33, then the survey will be terminated immediately] |
| 🔿 | 34. Preventive Medicine [If participant selects Q4 = 34, then the survey will be terminated immediately] |
| 🔿 | 35. Psychiatry |
| 🔿 | 36. Pulmonary Disease |
| 🔿 | 37. Radiation Oncology  [If participant selects Q4 = 37, then the survey will be terminated immediately] |
| 🔿 | 38. Radiology & Diagnostic Radiology  [If participant selects Q4 = 38, then the survey will be terminated immediately] |
| 🔿 | 39. Rheumatology |
| 🔿 | 40. Thoracic Surgery  [If participant selects Q4 = 40, then the survey will be terminated immediately] |
| 🔿 | 41. Urology |
| 🔿 | 42. Vascular & Interventional Radiology [If participant selects Q4 = 42, then the survey will be terminated immediately] |
| 🔿 | 43. Vascular Surgery [If participant selects Q4 = 43, then the survey will be terminated immediately] |
| 🔿 | 44. Other [If participant selects Q4 = 44, then the survey will be terminated immediately] |

Q5. How would you describe your primary practice setting (i.e., where you spend at least 75% of your time)?

*Please select one response.* [SINGLE-SELECT]

| 🔿 | 1. Primarily inpatient  [If participant selects Q5 = 1, then the survey will be terminated immediately] |
| --- | --- |
| 🔿 | 2. Primarily outpatient/office visits |
| 🔿 | 3. Emergency room [If participant selects Q5 = 3, then the survey will be terminated immediately] |

Q6. Which of the following best describes your primary facility/facilities?

*Please select ALL that apply.* [MULTI SELECT] [TERMINATE IMMEDIATELY IF CODES 4, 5 AND 7 ARE SELECTED REGARDLESS OF WHETHER 1 CODE FROM 1, 2, 3 or 6]

| ❒ | 1. Private practice |
| --- | --- |
| ❒ | 2. Community-based clinic |
| ❒ | 3. Academic institution |
| ❒ | 4. Military clinic |
| ❒ | 5. VA or government hospital |
| ❒ | 6. Hospital or hospital-owned (not VA or government) |
| ❒ | 7. Other |

Q9. Do you know the approximate staff time at your practice dedicated to work related to payer drug coverage policies, such as prior authorizations, step edits, and formulary restrictions? [SINGLE-SELECT]

| 🔿 | 1. Yes |
| --- | --- |
| 🔿 | 2. No [If Q9 = 2, then the survey will be terminated immediately] |

Q10a. You have met the screening criteria to participate in this 15–20-minute survey. All of the information you provide will be treated confidentially. Please acknowledge that you will provide accurate information and that you understand you will only be compensated based on completion of the full survey.

Would you like to participate? [SINGLE-SELECT]

| 🔿 | 1. Yes |
| --- | --- |
| 🔿 | 2. No [If Q10a = 2, then the survey will be terminated immediately] |

Section B: Participant and practice characteristics [SHOW TO RESPONDENTS AT THE TOP OF Q11]

[Please show Q11a and Q11b on one page]

Q11. a. For how many years have you been in your current position? [NUMERIC]

_________ [0 - 70] years

b. [If the participant is a physician (Q1 = 1)] For how many years have you been in practice since completing your medical training? [NUMERIC]

_________ [0 - 70] years [REQUIRE Q11b ≥ Q11a]

Q13. In what US state is your primary practice located?

[Drop down menu of all US states & DC]

Q16. What percentage of patients seen at your practice have the following types of insurance?

*Please enter only whole numbers between 0 and 100. Your total must add to 100%.*

[NUMERIC OR SINGLE-SELECT, SUM TO 100 AND MUST NOT BE LEFT AS MISSING & SHOW RUNNING TOTAL]

| 1. Private, commercial | __________% [0 - 100] [NUMERIC] |
| --- | --- |
| 2. Medicare (including Medicare Advantage, Part B, Part D, etc.) | __________% [0 - 100] [NUMERIC] |
| 3. Medicaid | __________% [0 - 100] [NUMERIC] |
| 4. Uninsured | __________% [0 - 100] [NUMERIC] |
| 5. Other (e.g., VA, military) | __________% [0 - 100] [NUMERIC] |
| 🔿 I do not know | |

[Show all parts of Q17 on one page]

Q17. [If participant is a physician (Q1 = 1)] What proportion of drug prescriptions at your practice fall under the following categories? [Add question to 2nd column header]

*Please enter only whole numbers between 0 and 100. Your total for parts a through d must add to 100%.* [NUMERIC, Q17a, Q17b, Q17c and Q17d MUST SUM TO 100% AND MUST NOT BE LEFT AS MISSING AND SHOW RUNNING TOTAL]

a. Generic drugs dispensed at a pharmacy

__________% [0 - 100]

[SHOW IF Q17a >0%] a1. What proportion of these are subject to payer drug coverage policies, such as formulary restrictions, prior authorizations, and step edits?

__________% [0 - 100]

b. Branded drugs dispensed at a pharmacy (not including specialty drugs)

__________% [0 - 100]

[SHOW IF Q17b >0%] b1. What proportion of these are subject to payer drug coverage policies, such as formulary restrictions, prior authorizations, and step edits?

__________% [0 - 100]

c. Specialty drugs dispensed at a pharmacy “Specialty” drugs are defined as high-cost oral or injectable drugs used to treat complex chronic conditions, often associated with high patient coinsurance (%).

__________% [0 - 100]

[SHOW IF Q17c >0%] c1. What proportion of these are subject to payer drug coverage policies, such as formulary restrictions, prior authorizations, and step edits?

__________% [0 - 100]

d. Physician-administered drugs

__________% [0 - 100]

[SHOW IF Q17d>0%] d1. What proportion of these are subject to payer drug coverage policies, such as formulary restrictions, prior authorizations, and step edits?

__________% [0 - 100]

Section C: Practice experience [SHOW TO RESPONDENTS AT THE TOP OF Q18]

[Show Q18a and Q18b and Q18c on same page]

Q18. [If the participant is a physician (Q1 = 1)] Please provide your best estimates concerning your patients only in a typical week:

a1. Number of patients [NUMERIC]: ___ [1 - 10,000]

a. Number of all drug prescriptions and administrations of physicians-administered drugs [NUMERIC]: ___ [1 - 10,000]

[If Q18a < 50, please show the following soft error/warning: “You entered <50 prescriptions and administrations per week. Please review your response and update if needed.”]

b. [If the participant is a physician (Q1 = 1)] Number of instances encountered by you and/or your staff for your patients in a typical week: [NUMERIC OR SINGLE-SELECT]

[If the participant is a physician (Q1 = 1), show the following instructions] *Please only enter instances related to your patients. Do not include the instances encountered by practice staff related to the patients of other physicians in your practice.*

[MAKE SURE AT LEAST 1 ROW HAS NUMERIC ENTRY, NOT ALL CAN BE “I do not know”. EACH ROW IN Q18b MUST BE <= q18a. If a row in Q18b is > Q18a, show the following error: “Please ensure your answer is less than or equal to [Q18 value], the total number of prescriptions and administrations that you entered.”]

| **Type of payer drug coverage policies** | **Volume per week** |
| --- | --- |
| 1. Formulary restrictions | ___ [0 - 10,000] [NUMERIC]  🔿 I do not know [EXCLUSIVE] |
| 2.Prior authorizations | ___ [0 - 10,000] [NUMERIC]  🔿 I do not know [EXCLUSIVE] |
| 3. Step edits | ___ [0 - 10,000] [NUMERIC]  🔿 I do not know [EXCLUSIVE] |
| 4. Other or unknown types of payer drug coverage policies | ___ [0 - 10,000] [NUMERIC]  🔿 I do not know [EXCLUSIVE] |

[Please show Q22a and Q22b on one page]

Q22. [If physician (Q1 = 1)] In a typical week:

a. How often did the cost to the patient for a drug cause you to spend additional time on your prescribing decisions (e.g., because of calls with the insurance company, discussion with the patient)? [SINGLE-SELECT]

🔿 Always (100% of the time)

🔿 Usually (~75% of the time)

🔿 Sometimes (~50% of the time)

🔿 Rarely (~25% of the time)

🔿 Never (0% of the time)

b. How often did you change your prescribing decision because patients were unable to afford a drug? [SINGLE-SELECT]

🔿 Always (100% of the time)

🔿 Usually (~75% of the time)

🔿 Sometimes (~50% of the time)

🔿 Rarely (~25% of the time)

🔿 Never (0% of the time)

Q23. [If physician (Q1 = 1)] How often do you decide against prescribing a particular drug due to the payer drug coverage policies that likely apply to the drug? [SINGLE-SELCT]

🔿 Always (100% of the time)

🔿 Usually (~75% of the time)

🔿 Sometimes (~50% of the time)

🔿 Rarely (~25% of the time)

🔿 Never (0% of the time)

Q24. In the last year, did your practice make any of the following investments to help handle tasks related to payer drug coverage policies? *Please select all that apply.*

[MULTI-SELECT; require respondents to select at least one option] [RANDOMIZE]

|  | 1. Allocate additional time from existing staff |
| --- | --- |
|  | 2. Pay overtime to hourly staff |
|  | 3. Hire additional staff |
|  | 4. Invest in additional office equipment or technology |
|  | 5. Invest in additional training for staff |
|  | 6. Invest in technology to support telehealth services |
|  | 7. Outsource some of the tasks previously performed by your staff |
| 🔿 | 10. Other, please specify __________ [ANCHOR] |
| 🔿 | 8. We have not made any investments [EXCLUSIVE] [ANCHOR] |

Q25. How often do patient visits at your practice last longer due to discussions with them about payer drug coverage policies? [SINGLE-SELECT]

[If the participant is a physician (Q1 = 1) show the following instructions] *Please answer based just on your own patient visits, do not include patient visits by other physicians or practice staff in your practice.*

🔿 Always (100% of the time)

🔿 Usually (~75% of the time)

🔿 Sometimes (~50% of the time)

🔿 Rarely (~25% of the time)

🔿 Never (0% of the time)

🔿 I do not know [ONLY SHOW IF ADMIN (Q1 = 2 or Q1 = 4)]

Q26. How often do payer drug coverage policies cause patients to come in for additional office visits, beyond what would have been required absent these payer drug coverage policies? [SINGLE-SELECT]

[If the participant is a physician (Q1 = 1) show the following instructions] *Please answer based just on your own patient visits, do not include patient visits by other physicians or practice staff in your practice.*

🔿 Always (100% of the time)

🔿 Usually (~75% of the time)

🔿 Sometimes (~50% of the time)

🔿 Rarely (~25% of the time)

🔿 Never (0% of the time)

🔿 I do not know [ONLY SHOW IF ADMIN (Q1 = 2 or Q1 = 4)]

Q27. How often do payer drug coverage policies cause patients to have additional lab tests or imaging (e.g., for monitoring, meeting minimum requirements for prescription), beyond what would have been done absent these payer drug coverage policies? [SINGLE-SELECT]

[If the participant is a physician (Q1 = 1) show the following instructions] *Please answer based just on your own patients, do not include patients of other physicians or practice staff in your practice.*

🔿 Always (100% of the time)

🔿 Usually (~75% of the time)

🔿 Sometimes (~50% of the time)

🔿 Rarely (~25% of the time)

🔿 Never (0% of the time)

🔿 I do not know [ONLY SHOW IF ADMIN (Q1 = 2 or Q1 = 4)]

Q28. [If physician (Q1 = 1)] In a typical week, please select all of the following that occur because of time you spend related to payer drug coverage policies. [MULTI-SELECT; require respondents to select at least one option] [RANDOMIZE]

*Please select all that apply.*

|  | 1. I had to schedule fewer patient visits |
| --- | --- |
|  | 2. I had to reschedule patient visits |
|  | 3. I had to spend extra time working before/after office hours, or over the weekend |
|  | 4. I had to cut down the amount of time I spent on other administrative tasks |
| 🔿 | 5. None of the above [EXCLUSIVE] [ANCHOR] |

Section D: Physician perceptions [SHOW TO RESPONDENTS AT THE TOP OF Q32]

Q32. [If physician (Q1 = 1)] How often do you feel that each of the following payer drug coverage policies are aligned with clinical evidence/your best professional judgement? [SINGLE-SELCT FOR EACH ROW]

[To hide rows with volume of 0 from Q18b]

|  | Never (0% of the time) | Rarely (~25% of the time) | Sometimes (~50% of the time) | Usually (~75% of the time) | Always (100% of the time) | I do not know |
| --- | --- | --- | --- | --- | --- | --- |
| 1.Formulary restrictions | 🔾 | 🔾 | 🔾 | 🔾 | 🔾 | 🔾 |
| 2.Prior authorizations | 🔾 | 🔾 | 🔾 | 🔾 | 🔾 | 🔾 |
| 3.Step edits | 🔾 | 🔾 | 🔾 | 🔾 | 🔾 | 🔾 |

Q33. [If physician (Q1 = 1)] For payer drug coverage policies that would be considered medically justified, which of the following do you believe these policies should be based on?

*Please select all that apply.*

[MULTI-SELECT; require respondents to select at least one option] [RANDOMIZE]

|  | 1. FDA labels |
| --- | --- |
|  | 2. Clinical guidelines and medical society guidelines (e.g., NCCN guidelines, compendia listings) |
|  | 3. Real world outcomes evidence |
|  | 4. Peer-reviewed publications of clinical trial data (e.g., trial results, meta-analyses, systematic reviews) |
|  | 5. Payer’s discretion |
| 🔿 | 6. None of the above [EXCLUSIVE] [ANCHOR] |
